# Expression of a Tuberculosis-Associated Immunogenic Protein in *Escherichia coli*

**DOI:** 10.3390/life15091472

**Published:** 2025-09-19

**Authors:** Gizem Kılıç, Burcu Saygıner, Muhammed Yusuf Yılmaz, Bilge Suyolcu Albayrak, Neda Tatlıoğlu, Ayça Tan, Tanil Kocagoz, Nihan Ünübol, Erkan Mozioğlu

**Affiliations:** 1 Department of Medical Biotechnology, Institute of Health Sciences, Acibadem University, 34752 İstanbul, Türkiye; 2 Department of Medical Microbiology, Faculty of Medicine, Acibadem University, 65719 İstanbul, Türkiye; 3 Medical Laboratory Techniques, Vocational School of Health Services, Acibadem University, 65719 İstanbul, Türkiye

**Keywords:** *M. tuberculosis*, MPT64, recombinant protein, codon optimsation, LIC

## Abstract

It is estimated that one in four people worldwide carries *Mycobacterium tuberculosis* bacteria. MPT64 is a protein exclusively secreted by *Mycobacterium tuberculosis complex* (MTC) bacteria. It serves as a crucial diagnostic marker and plays a role in the bacterium’s survival by modulating the host immune response. Consequently, the development of innovative diagnostic tools based on MPT64, as well as the production of high-purity MPT64 protein to support research on tuberculosis pathogenesis and the advancement of novel therapeutic strategies, is of great importance. In this study, optimization experiments were conducted to produce this protein in *E. coli* with high yield and purity. First, a gBlock was designed by codon optimization and then cloned into a plasmid vector using the LIC method. For more efficient production, *E. coli* BL21(DE3)-R3-pRARE2 strain, which carries rare tRNAs for rare codons, was used as the host. Five different culture media were tested to maximize protein production, with the highest yield obtained in eBHI medium. The resulting protein yield was 4.9 mg/L. To the best of our knowledge, this study provides the most detailed information on the recombinant production and characterization of MPT64 to date. Therefore, these results contribute important data for future studies on the MPT64 protein.

## 1. Introduction

Tuberculosis (TB) remains a serious threat to humanity worldwide [1,2]. The main reason for this is the high contagiousness of the *Mycobacterium tuberculosis* bacteria that causes tuberculosis [1,2]. According to the World Health Organization (WHO), one in every four people worldwide carries the bacterium [1,2]. In some of these people, the bacterium remains inactive; this is known as latent tuberculosis [2]. Active tuberculosis develops in infected individuals if the body is unable to stop the growth of the causative bacteria [2]. These bacteria, which are among the leading infectious agents that cause the highest number of deaths, must be taken more seriously, especially in today’s world of rapidly increasing global mobility. In this regard, the WHO Global Tuberculosis Program aims to achieve a world free of tuberculosis [1].

In the fight against tuberculosis worldwide, vaccination is one of the main methods used, especially in countries with a high incidence of tuberculosis. Bacillus Calmette-Guérin (BCG), an attenuated strain of *M. bovis*, which causes tuberculosis in cattle, is used as a vaccine agent [3]. The BCG bacterium is a representative of the same group as *M. tuberculosis*, as well as other mycobacteria such as *M. africanum*, *M. bovis*, *M. caprae*, *M. canettii*, *M. microti*, and *M. pinnipedii*, collectively forming the MTC [3]. All these bacteria are genotypically very similar but differ in certain genomic regions. One such region, Region of Difference 2 (RD2), encodes the MPT64 protein [4,5]. The first BCG vaccine was BCG-Pasteur, and this lineage is the source of BCG vaccines in many other countries [4,5]. This BCG strain, which is a non-infectious strain of *M. bovis*, produces the MPT64 protein just like *M. tuberculosis* [4,5]. However, over time, mutations have occurred in BCG strains worldwide, even among those derived from the same lineage, and in some cases, the RD2 region has been deleted. As a result, these BCG strains are unable to produce the MPT64 protein [4,5]. The lack of the MPT64 protein has been argued to be possibly related to the failure of some BCG vaccines to induce an adequate immune response [4].

Taking all this into account, why is the MPT64 protein important? MPT64 is a protein specifically secreted by members of the MTC, which functions as an important diagnostic marker and as a factor that contributes to bacterial survival by modulating the host immune response. One of its advantages is its detectability in body fluids such as blood and urine, which are easier to obtain and process than more challenging samples like sputum [6,7]. A positive MPT64 test result suggests tuberculosis infection, particularly since some BCG strains lack the RD2 gene responsible for encoding this protein. Similarly, this protein is a common product of mycobacteria within the tuberculosis complex but is absent in non-tuberculous species [4,5]. Thus, detection of MPT64 serves as a reliable method for distinguishing the MTC from non-tuberculous mycobacteria (NTM) [4,5]. Another important consideration regarding the MPT64 protein is its potential role as a therapeutic target [8]. For example, *M. tuberculosis* is taken up by host macrophages through phagocytosis, a defense mechanism intended to eliminate the bacteria [9]. Normally, macrophages may undergo apoptosis to destroy internalized pathogens. However, *M. tuberculosis* has evolved strategies to subvert this process, thereby enhancing its own survival [9]. One of these mechanisms involves the use of MPT64, which has been shown to reduce macrophage apoptosis [9]. In this context, the importance of MPT64 in both tuberculosis diagnosis and treatment becomes evident [6,7,8,9]. Consequently, there is a strong need for the development of novel diagnostic tools based on MPT64, as well as the production of high-purity MPT64 protein to facilitate research on tuberculosis pathogenesis and the development of new therapeutic approaches.

The MPT64 protein can potentially be obtained by purification directly from *M. tuberculosis* cultures. However, given the bacterium’s replication time of 24–30 h, it is nearly impossible to obtain large amounts of protein, especially since MPT64 represents only one of 1314 proteins expressed in the bacterial culture [10]. Additionally, the purity of the protein from such a culture of pathogenic bacteria would raise serious concerns, especially if these proteins were to be used in in vivo applications. Considering these limitations, recombinant production of MPT64 protein becomes a more favorable approach. There are few studies in which *E. coli* and *M. smegmatis* bacteria were used as host cells for the production of recombinant MPT64 protein. As a result of cloning into *M. smegmatis*, codon optimization was not required due to its phylogenetic similarity to *M. tuberculosis*. In these studies, the protein was secreted into the culture medium and could be purified directly from the supernatant using nickel affinity chromatography [11]. However, production required three-day cultures due to the slower growth rate of *M. smegmatis* compared to *E. coli*. Moreover, as both are mycobacteria, special attention was needed to ensure the removal of LAM or other antigenic residues that could interfere with downstream applications. There are also studies involving recombinant expression in *E. coli*, a faster-growing bacterium [12,13,14,15,16,17,18,19]. In these cases, DNA from MTC strains was used as the gene source. However, the codon similarity between the MPT64 gene from *M. tuberculosis* and the codon usage preference of *E. coli* is approximately 77%, which limits expression efficiency and yield. One study in the literature employed a synthetic gene [14] but did not report codon optimization; instead, only expression conditions were tested, and purified protein was not demonstrated. Clearly, further research is necessary to obtain MPT64 protein in high yield and purity. This is particularly important given the protein’s potential as a theranostic molecule in tuberculosis.

In this study, for the expression of the MPT64 protein, codon optimization was initially performed to adapt codon usage to that of *Escherichia coli*, and a synthetic gene block was designed accordingly. The optimized gene was expressed in *E. coli* BL21(DE3)-R3-pRARE2 cells, a strain engineered to supply tRNAs for rare codons (RARE), enhancing translational efficiency. To facilitate the cloning process, a ligation-independent cloning (LIC) strategy was employed. Subsequently, optimization studies were conducted to maximize protein yield using not only standard Luria-Bertani (LB) medium but also alternative culture media, including Mueller Hinton Broth (MHB), Brain Heart Infusion (BHI) broth, and modified BHI formulations developed in this study.

As a result of these combined strategies, the MPT64 protein was obtained in high purity and yield. Therefore, the findings of this study provide valuable insights for future research involving recombinant MPT64 protein.

## 2. Materials and Methods

### 2.1. Materials

Gene block (gBlock) and primers were obtained from IDT Inc., Coralville, IA, USA 5x PCR Master Mix (#RP02-II-400) and Plasmid Purification Kit (#DP01) were bought from GeneMark, Taizhou, China. T4 DNA Polymerase (#70099-M) and Phusion High-Fidelity DNA Polymerase (#M0530S) were bought from Sigma (Novagen), Darmstadt, Germany, and NEB, Hitchin, UK. SspI enzymes were bought from NEB. *E. coli* BL21(DE3)-R3-pRARE2 was obtained from Addgene (#26242), London, UK. LB (Miller’s formulation) was from Sigma, Darmstadt, Germany; MHB and BHI were from Across Bio, Shanghai, China. Urea, Guanidinium HCl (#G211), and Nickel beads (#R202) were bought from Goldbio, St. Louis, MO, USA. Omni-Ruptor 4000 (Kennesaw, GA, USA) was used for ultrasonic homogenization. Capilla TB-Neo strip test (#CATB0870), Shizuoka, Japan, was used for antibody-based confirmation of MPT protein.

### 2.2. Methods

**Figure 1:**
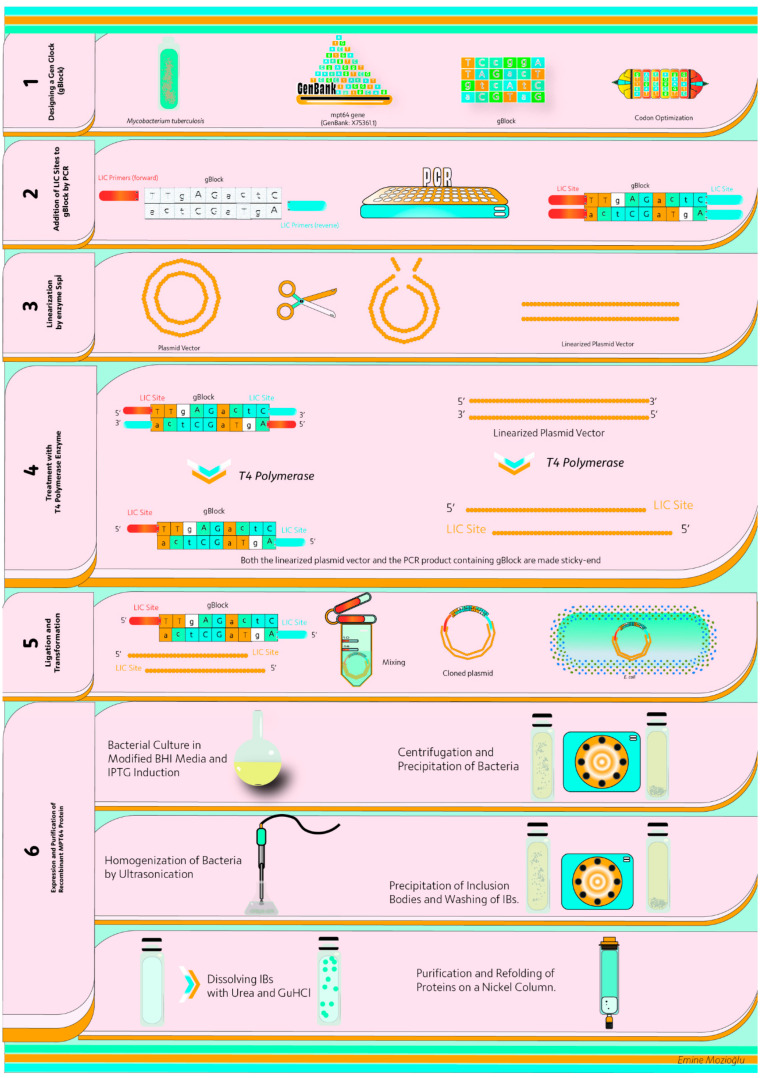
Graphical abstract showing all steps followed in this study. (1) gBlock Design; (2) Addition of LIC sites to gBlock; (3) Linearization of plasmid vector by SspI; (4) Production of sticky ends by T4 DNA Polymerase; (5) Ligation and transformation; (6) Production and purification of MPT64 protein. The graphic was designed by Emine Mozioğlu.

All the steps followed in this study are briefly illustrated in Figure 1.

### 2.3. Design of gBlock and LIC

The MPT64 protein sequence of *M. tuberculosis* was obtained from NCBI (NP_216496.1). In order to determine the most likely DNA sequences that allow this protein to be produced in *E. coli*, codon optimization was performed. The *pET His6 TEV LIC* vector was used as the cloning and expression vector, which was developed by Scott Gradia and deposited in Addgene (# 29653). Cloning was performed by adding LIC sites to the MPT64 gene by PCR [20]. Studies related to primer designs and PCR optimization are provided in detail in the Supplementary Materials section. Based on optimization results, the PCR reaction mixture was prepared as follows: a final concentration of 0.2 mM dNTP, 0.5 mM primers and 0.02 U Phusion High-Fidelity DNA Polymerase, DMSO of 5%, 20 ng gBlock in 1X GC buffer (NEB). PCR conditions were as follows: 98 °C for 3 min for initialization, 98 °C for 10 s, 72 °C for 90 s for 30 cycles, and then 72 °C for 3 min. Then, for ethanol precipitation, the amplicon was mixed with one-tenth of its own volume of 3M NaOAc (pH 5.2) and approximately 3 volumes of cold ethanol, with MgCl_2_ added to a final concentration of 1 mM. The mixture was kept on ice for 15 min. After centrifugation at 13,000× *g* for 15 min, the supernatant was discarded and the tubes were dried by leaving them open. Amplicons were dissolved in 30 µL of water and stored at −20 °C until the next step.

Both plasmid vectors and gBlock-PCR products were made sticky-ended by the T4 polymerase enzyme [20]. Since the T4 polymerase enzyme shows exonuclease activity in the presence of only a single nucleotide, PCR products were treated with dCTP, while plasmid vectors were treated with dGTP. The reaction was as follows: 22 °C for 30 min and 75 °C for 20 min. Then, both products were mixed in different ratios and kept at 70 °C for 1 min, 22 °C for 5 min, and on ice for 30 min [20]. Transformations were performed by applying heat shock to bacteria that were made competent using the CaCl_2_ method as explained in detail in the Supplementary Materials, and sequences were confirmed by Sanger sequencing.

### 2.4. Optimization of Requirements for Recombinant Protein Expression

To optimize protein expression, several parameters were systematically investigated. First, the impact of different culture media on expression levels was assessed by comparing standard LB, MHB, and BHI media, along with BHI media supplemented and enriched through modifications developed in this study. The influence of IPTG induction was also examined to determine its role in enhancing protein yield. Additionally, the effect of bacterial density at the time of induction, specifically at optical densities (OD_600_) of 0.6 and 2.5, was evaluated. Time-dependent expression efficiency was another variable explored to assess how extended or shortened induction periods influenced total protein production. Finally, each of these conditions was analyzed in terms of their influence on the solubility of the expressed protein, with particular attention to whether the protein was produced in a soluble or insoluble form.

Full details of the experiments related to protein expression optimization are provided in the Supplementary Materials. Briefly, the optimization was first performed by comparing the effects of five different medium ingredients on protein expression efficiency. After bacteria were grown as a starter culture overnight, the next day, they were transferred into fresh culture media. Once the optical density (600 nm) reached 0.5, IPTG (at a final concentration of 0.1 mM) was added to the culture, and 100 µL of the growing bacterial culture was taken at different times. The samples without IPTG were also taken as controls. Then, bacteria were precipitated by centrifugation at 16,000× *g* for 5 min, and the supernatant was removed. Samples were run on a 12% SDS gel, and gels were stained with the CBB-HCl method [21]. The molecular weight of the MPT64 protein with His and TEV sites is exactly 26,745 Daltons.

After optimizing the expression conditions, the effects of bacterial density on protein yield and the production of soluble/insoluble proteins were investigated. For this purpose, the bacterial density prior to IPTG induction was set at two different levels (OD 600 nm at 0.5 and 2.5), and at the end of expression, bacteria were precipitated by centrifugation at 10,000× *g* for 10 min and then suspended in 5 mL of 10 mM Tris-HCl buffer (pH 8.0) containing 500 µg lysozyme and 100 µg DNase I. The sample was kept at room temperature for 30 min by shaking, and then ultrasonic treatment was applied for 5 min. After centrifugation at 10,000× *g* for 1 h, the supernatant was removed, and the pellet was dissolved in 5 mL of 7M urea for 40 min at room temperature. Both the supernatant and the precipitate were analyzed using 12% SDS-PAGE.

### 2.5. Production and Purification of Recombinant MPT64 Protein

In order to obtain large amounts of pure recombinant MPT64 protein using optimized eBHI, the following studies were carried out:

### 2.6. Production of the Inclusion Body

Inclusion body production was performed by modifying the protocols in the literature [22,23,24]. Twenty milliliters of starter bacterial culture was incubated in eBHI medium overnight at 37 °C by shaking at 220 rpm. The next day, 400 mL of fresh culture was prepared. Once OD_600_ was 2.5, IPTG was added at a final concentration of 1 mM, and the culture was incubated overnight at 37 °C by shaking at 220 rpm. The 400 mL bacterial culture was centrifuged at 10,000× *g* for 10 min, and the supernatant was discarded. The bacterial pellet was suspended in 40 mL of 100 mM Tris-HCl (pH 8.0) buffer containing 1mM EDTA. Two hundred microliters of 20 mg/mL lysozyme and 40 µL of 20 mg/mL DNase I were added to the suspension and incubated for 30 min at room temperature. After ultrasonication (200 watts of power) for 5 min, the tubes were centrifuged at 10,000× *g* for 1 h at +4 °C. After removing the supernatant, the pellet was suspended in 100 mM Tris-HCl (pH 8.0) buffer containing 1 mM EDTA and the supernatant was removed by centrifugation at 4 °C at 10,000× *g* for 1 h. This washing step was repeated 7 times, and proteins were analyzed using 12% SDS-PAGE.

The final inclusion body was exposed to ultrasonic sound for 1 min in 10 mL of 100 mM potassium buffer (KH_2_PO_4_-K_2_HPO_4_, pH 7.4), and 20 μL of 20 mg/mL DNase I was added and incubated at room temperature for 1 h. After centrifugation at 10,000× *g* for 15 min, the supernatant was removed, and the pellet was suspended in 10 mL of 100 mM potassium buffer (KH_2_PO_4_-K_2_HPO_4_, pH 7.4). Then, 10 μL of 20 mg/mL DNase I was added and incubated for 15 min at room temperature. After centrifugation at 10,000× *g* for 15 min, the supernatant was removed, and the pellet was suspended in 10 mL of 100 mM potassium buffer (KH_2_PO_4_-K_2_HPO_4_, pH 7.4). Ten milliliters of glycerol was added, and after mixing, it was aliquoted in 1 mL each and kept at −80 °C for further use.

### 2.7. Purification of the MPT64 Protein

To 1 mL aliquoted inclusion body sample, a final concentration of 1 mM CaCl_2_, 2.5 mM MgCl_2_, and 100 µg DNase I were added and incubated overnight at room temperature. To dissolve the protein, 2 mL of solubilization buffer (7 M urea, 6 M Guanidinium HCl (Gu-HCl), 5 mM DTT, 10 mM Tris-HCl pH 8.0 at final concentration) was added. After centrifugation at 4 °C at 20,000× *g* for 1 h, the supernatant was transferred to a clean tube, and 500 mM NaCl and 20 mM imidazole were added at final concentration. The protein sample was passed through a nickel column, and gravity flow was used. The column was washed by reducing both urea and Gu-HCl concentrations gradually (7, 6, 5, 4, 3, 2, 1, and 0 M) to allow the protein to refold on the column. Proteins were then eluted by the buffer containing 250 mM and 500 mM imidazole, three times each. Recovery samples were analyzed using 12% SDS-PAGE.

In order to eliminate impurities in the elution step, different imidazole concentrations were tried. For this purpose, the inclusion body was dissolved as described above and refolded on the column using nickel beads. Recovery was performed by using elution buffer containing 100 mM imidazole three times, then 200, 250, and 350 mM once each, and finally 500 mM imidazole twice. Recovered proteins were analyzed using 12% SDS-PAGE.

For further optimization, the elution step was performed 4 times with 100 mM imidazole followed by 125, 150, 175, 250, and 500 mM imidazole once each. The purity of the proteins obtained was analyzed using 12% SDS-PAGE. Recovered protein samples were pooled and concentrated by 50 kDa ultrafiltration (Amicon, Millipore, Darmstadt, Germany), and then buffer exchange was performed in 10 mM Tris-HCl (pH 8.0) buffer. The protein sample was analyzed using 12% SDS-PAGE and quantified by Bradford assay.

### 2.8. Characterization of the MPT64 Protein

#### 2.8.1. Lateral Flow Assay for Confirmation of Recombinant MPT64 Protein

Purified recombinant MPT64 protein was confirmed using a lateral flow assay (SD Bioline TB Ag MPT64 Rapid) based on the use of specific antibodies. The assay was performed as recommended by the manufacturer. Briefly, the protein was added to the sample loading well, and the visualization of red bands on both the control line (C) and test line (T) was considered a positive result confirming the presence of the MPT64 protein.

#### 2.8.2. Circular Dichroism (CD) Analysis

CD measurements were performed using a Jasco J-815 instrument as described in the literature [14,25]. The scanning between 195 and 300 nm wavelengths was performed five times and averaged.

#### 2.8.3. Determination of Multimeric Forms of Recombinant MPT64 Protein

In order to determine the multimeric forms of recombinant MPT64 protein, a method based on fractionation of the protein according to its size was first used. Therefore, the presence of refolded proteins in 30K and 50K filters (Amicon, Millipore) was examined to determine the fraction in which they could be found. For this purpose, proteins were first passed through a 30K filter, and it was determined whether the protein remained on the filter or passed through to the bottom by 12% SDS PAGE. Then, the proteins were passed through a 50K filter. The sample remaining on top of the filter was transferred to a clean tube for analysis. The bottom sample was passed through a 30K filter. Both the top and bottom of the 30K filter were similarly collected for analysis. All samples were analyzed using 12% SDS-PAGE.

## 3. Results

### 3.1. Design of gBlock

Codon optimization was performed to determine the most likely DNA sequences for the production of this protein in *E. coli* (Figure S1).

### 3.2. Optimization of PCR

Three different binding temperatures were tested in the amplification of gBlock with LIC primers. According to these results, non-specific amplification products were obtained (Figure S2).

In order to remove non-specific amplification products, PCR was performed by adding DMSO at different concentrations. According to the agarose gel results of the amplification products, non-specific bands were eliminated in the presence of 5% (*v*/*v*) DMSO (Figure S3). PCR was repeated in the presence of 5% (*v*/*v*) DMSO, and amplicons were purified by ethanol precipitation.

### 3.3. LIC and Transformation

Plasmid vectors were cut by the SspI enzyme to linearize them (Figure S4).

After linearization of plasmid vectors with SspI, both vector and PCR products were made sticky-ended by T4 DNA polymerase. Both products were mixed in different ratios for ligation and transferred to DH5α strain. Plasmids were purified from bacteria carrying the MPT64 gene and confirmed by Sanger sequencing.

After confirmation by Sanger sequencing, plasmids were transferred to *E. coli* BL21(DE3)-R3-pRARE2 strains for expression, and colonies were screened by PCR (Figure S5).

### 3.4. Optimization of Requirements for Recombinant Protein Expression

The amino acid sequence of the MPT64 protein is shown in Figure S6. The efficiency of recombinant MPT64 expression in media with different ingredients was determined based on hourly samples run on SDS-PAGE.

#### 3.4.1. LB Medium

LB medium, which is the most commonly used medium for protein production, showed very low gene expression both within the first 5 h following IPTG stimulation and at the end of 24 h. Expression was also low in controls without IPTG, but it was higher than the culture with IPTG (Figure S7).

#### 3.4.2. MHB Medium

Spectrophotometric measurements (OD_600_) showed that the cloned bacteria grown in MHB had very low turnover rates. Protein production was similar to that in LB medium after 24 h. Protein production was observed in both IPTG-stimulated and non-stimulated samples at the end of 24 h. Also, unlike LB, it was observed that the amount of protein started to increase after 4 h (Figure S8).

#### 3.4.3. BHI Medium and Modified BHI Media

After 24 h, the amount of protein obtained (both in the presence and absence of IPTG) in BHI medium was similar to that obtained with MHB medium. However, the increase in the amount of protein in samples taken during the first five hours was much less than in MHB and LB (Figure S9).

In comparison with BHI medium, the total mass of bacteria and total protein content increased significantly in BHI medium supplemented with MgCl_2_ and glycerol (sBHI). At the fifth hour, the sample without IPTG produced more protein than the sample with IPTG, while at the end of 24 h, the sample with IPTG produced more protein (Figure S10).

Besides MgCl_2_ and glycerol, the addition of peptone and tryptone to the BHI medium resulted in a dramatic increase in the total mass of the bacteria (according to spectrophotometric measurements). Protein amounts increased hourly, and similar to the sBHI medium, more protein was produced in the sample without IPTG than in the sample with IPTG at the fifth hour, while the sample with IPTG produced more protein at the end of 24 h (Figure S11).

As a result of optimization experiments with different media, it was observed that overall the production of the protein was similar in all the media tested. The bacterial biomass was the lowest in MHB medium while it was the highest in eBHI. Therefore, eBHI medium was used for the production of recombinant MPT64 proteins.

#### 3.4.4. Soluble and Insoluble Fractions of the Expression

**Figure 2:**
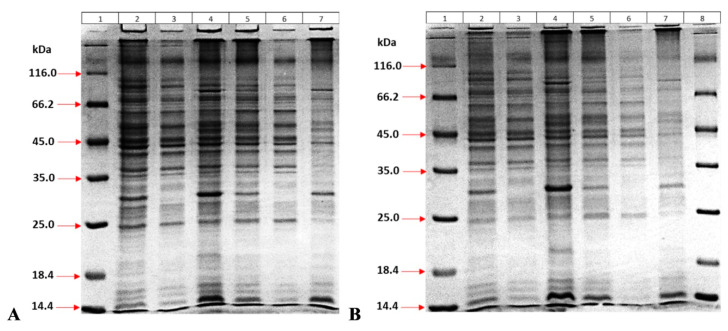
Protein profile at (**A**) OD_600_ = 0.6; (**B**) OD_600_ = 2.5 bacterial density. (1) Marker (Fermentas); with IPTG (2) Homogenized whole bacteria; (3) Supernatant; (4) Pellet: and without IPTG (5) Homogenized whole bacteria; (6) Supernatant; (7) Pellet; (8) Marker.

Protein expression was repeated in eBHI medium. Here, the effect of bacterial density on expression was investigated. Therefore, two different bacterial densities were studied before induction with IPTG: OD_600_ = 0.6 (Figure 2A) and 2.5 (Figure 2B). This resulted in a higher amount of protein for the culture at OD_600_ 2.5 compared to the other. Furthermore, the form of the protein produced (soluble or insoluble) in both cases was investigated. The gel results showed that recombinant MPT64 proteins were produced in insoluble form (Figure 2).

#### 3.4.5. Production of the Inclusion Body

**Figure 3:**
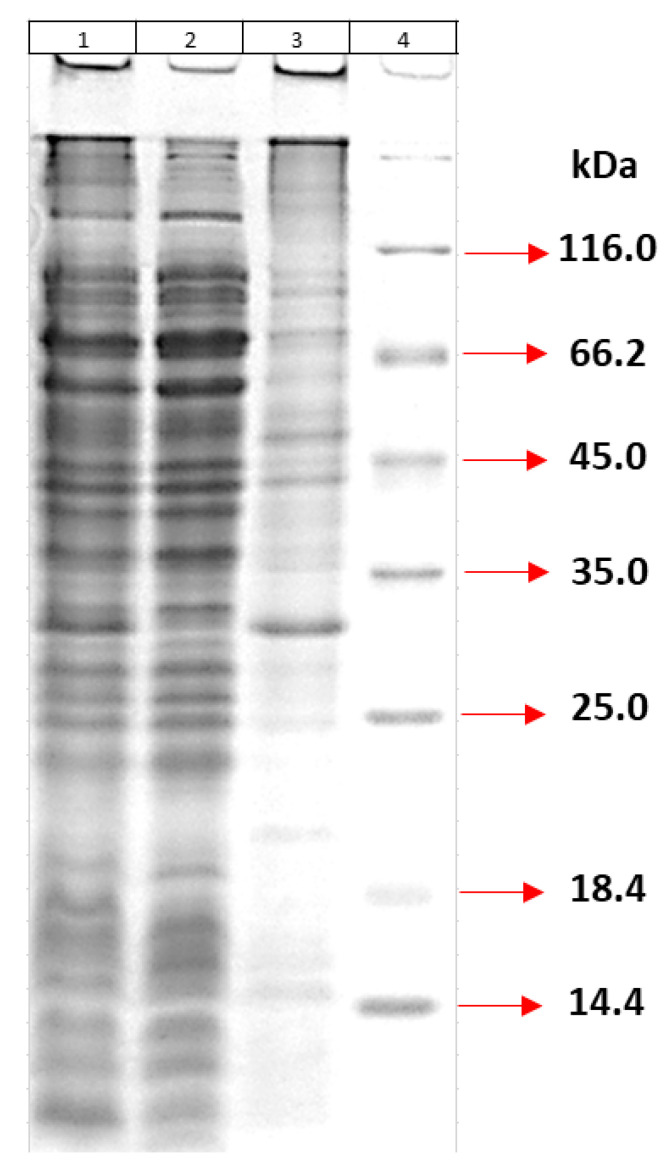
Protein profile. (1) Homogenized whole bacteria; (2) Supernatant; (3) Pellet obtained after washing seven times; (4) Marker.

For the production of large amounts of recombinant MPT64 protein, bacterial cells were cultured in optimized eBHI medium and homogenized by ultrasonication. Proteins were produced as inclusion bodies and then analyzed by 12% SDS-PAGE after 7 repeated washing steps (Figure 3). According to the gel results, most of the residual proteins were removed by washing, but some impurities remained.

#### 3.4.6. Optimization of the Purification Method

**Figure 4:**
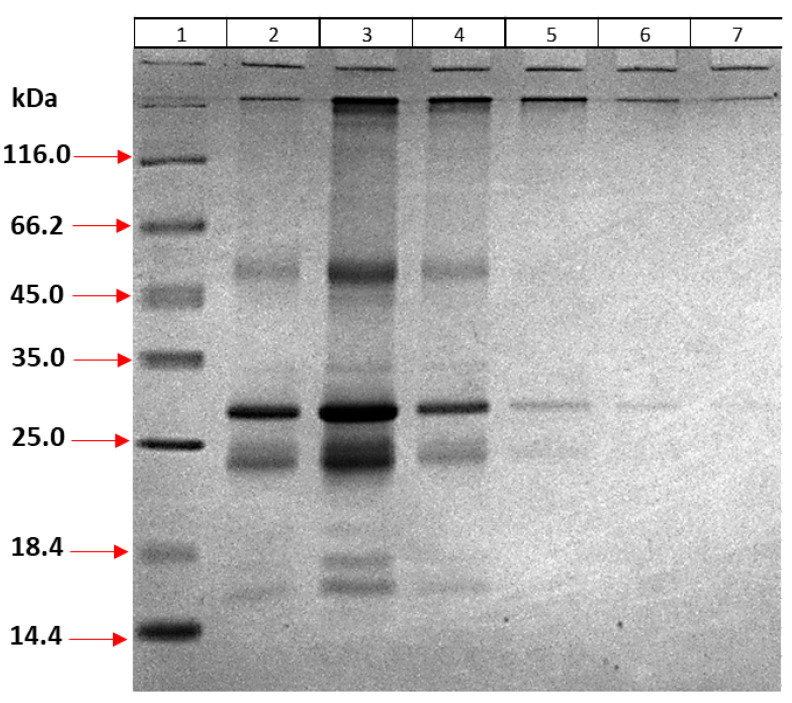
Optimization of purification steps. (1) Marker; Eluted proteins in buffer including (2–4) 250 mM imidazole and (5–7) 500 mM imidazole.

Recombinant MPT64 protein, which can be obtained as inclusion bodies, was first solubilized with denaturing agents such as urea and Gu-HCl. Then, the proteins were bound to nickel beads through the 6x histidine tail, and other proteins were removed. The concentrations of the denaturants were gradually reduced, allowing the proteins to refold on the column. For elution, 250 mM and 500 mM imidazole were applied, and the proteins were analyzed by 12% SDS-PAGE (Figure 4). According to the gel results, the MPT64 protein was recovered with 250 mM imidazole, but impurities were present. The MPT64 protein was still found in the 500 mM imidazole samples and was relatively more pure, but in smaller quantities.

In order to obtain the MPT64 protein with higher purity, the imidazole concentrations in the elution buffer were optimized, and recovered samples were analyzed by 12% SDS-PAGE (Figure S12). According to the gel results, the MPT64 protein could be recovered more purely at imidazole concentrations between 100–200 mM. The effect of a narrower imidazole concentration range on the purity of the recovered proteins was tested. According to the SDS-PAGE results, it was observed that concentration differences of 25 mM in the range of 100–175 mM were more effective in recovering the protein with higher purity from the nickel column (Figure S13). The recovered proteins were concentrated and buffer-exchanged with 10 mM Tris-HCl (pH 8.0) buffer. The amounts of proteins were determined by Bradford assay, and samples were analyzed by 12% SDS-PAGE (Figure S14). The amount of protein obtained from 1 mL inclusion body was measured as 98 µg by Bradford assay. This shows that a total of 4.9 mg/L of recombinant MPT64 protein can potentially be obtained by this method. The purity of the MPT64 protein was estimated to be 94.7% using ImageJ.

#### 3.4.7. Characterization of the MPT64 Protein

##### Lateral Flow Assay

**Figure 5:**
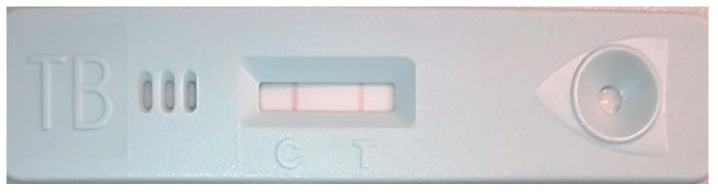
Lateral flow assay. (C) Control line; (T) Test line confirming recombinant MPT64 protein.

The purified recombinant MPT64 protein was confirmed by using an antibody-based lateral flow assay (Figure 5). In this assay, the test line appears red, indicating the presence of MPT64 protein. In summary, using antibodies specific to the MPT64 protein, the identity of the proteins obtained in the study was confirmed.

##### CD Analysis

**Figure 6:**
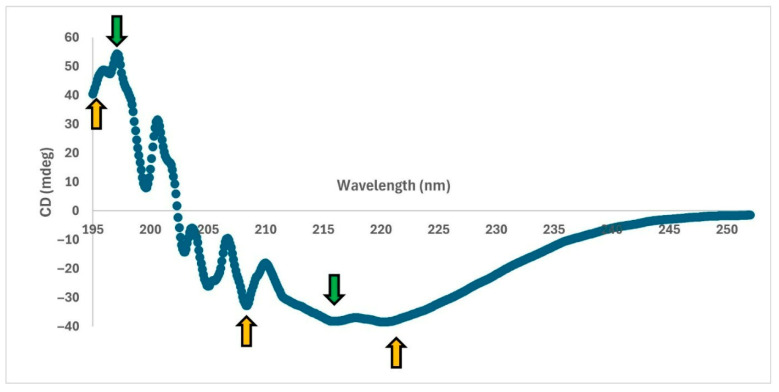
CD Spectrum (195–250 nm). Yellow arrows: α-helix structure; Green arrows: β-sheet structure.

CD measurements were performed between 195–300 nm wavelengths, and the average of five repeated scans was graphed (Figure 6). In CD analysis, which is used to determine the secondary structures of proteins, spectral features between 190 nm and 230 nm are evaluated [25]. Accordingly, α-helical structures exhibit a characteristic double peak at 208 nm and 222 nm and a stronger maximum around 191–193 nm, while β-sheet structures are characterized by a minimum near 215 nm and a maximum near 198 nm [25]. As expected, α-helical and β-sheet structures were observed in the protein, which was consistent with other CD results given in the literature for MPT64 [14].

##### Determination of Multimeric Forms of Recombinant MPT64 Protein

**Figure 7:**
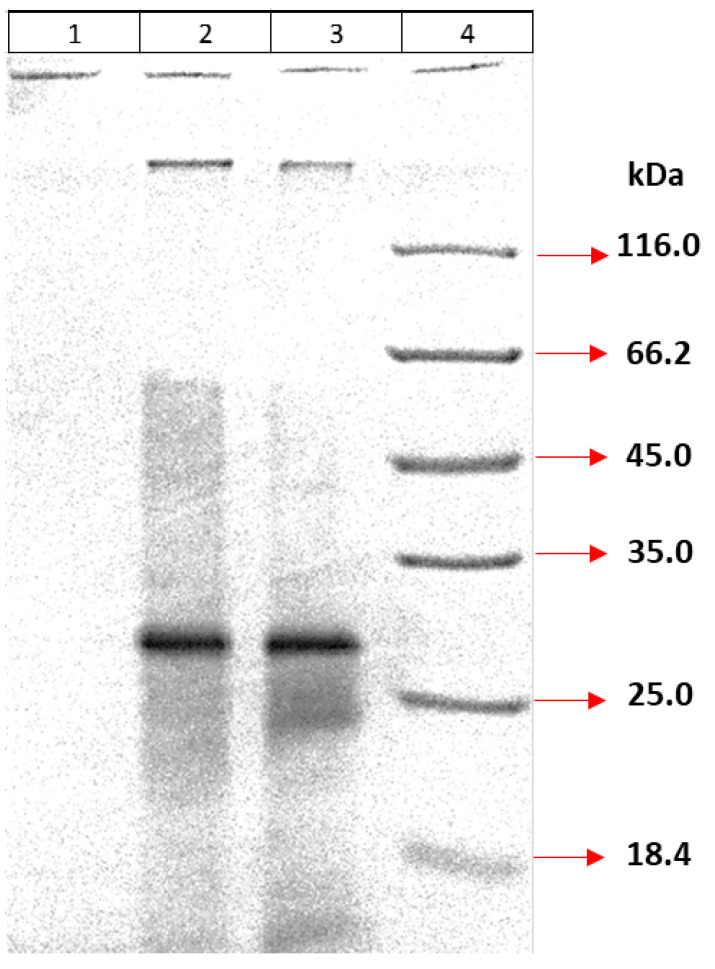
Ultrafiltration (30K) of recombinant MPT64 protein. (1) Sample passing through the filter after ultrafiltration; (2) Sample remaining on top of filter; (3) Sample before ultrafiltration; (4) Marker.

Multimeric forms of refolded proteins were determined based on size using 30K and 50K ultrafiltration tubes (Amicon, Millipore, Germany). First, the proteins were passed through a 30K filter. It was observed that the entire protein remained on top of the filter, while no protein was detected below (Figure 7).

**Figure 8:**
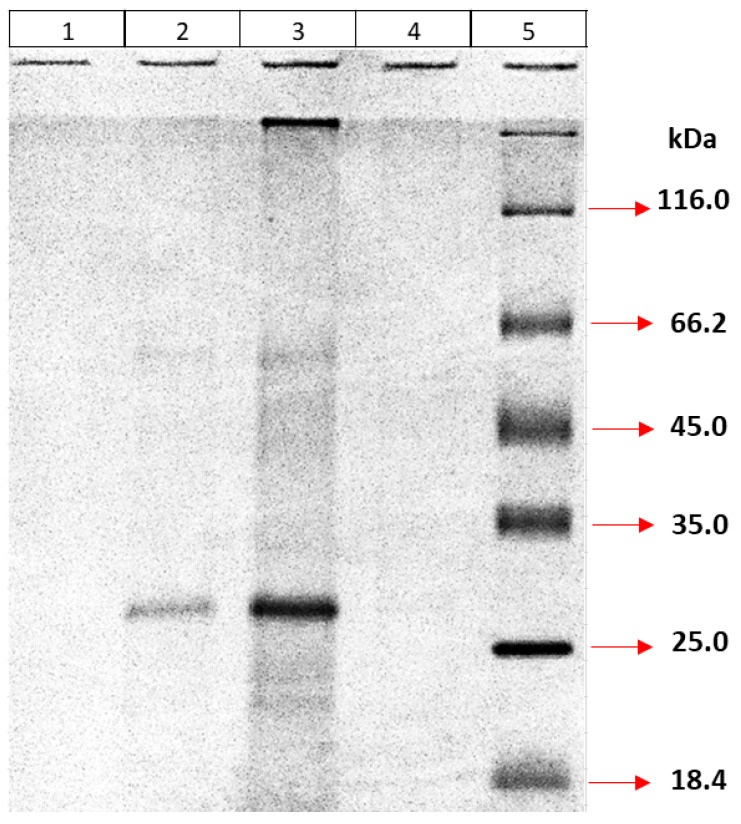
Ultrafiltration of recombinant MPT64 protein. (1) Marker; (2) Sample passing through the filter (50K); (3) Sample remaining on top of filter (50K); (4) Sample passing through the filter (30K); (5) Sample remaining on top of filter (30K).

A similar experiment was then repeated with a 50K ultrafiltration tube. Samples remaining on top of the filter or passing down the filter were analyzed. Furthermore, the sample going down through the 50K filter was filtered again with a 30K filter, and both the samples remaining on top of the 30K filter and the samples going down through the filter were analyzed using 12% SDS-PAGE (Figure 8). According to the results of the ultrafiltration experiments, the majority of the protein remained on top of the 50K filter, and a small portion was found above the 30K filter. No protein passing through the 30K filter could be detected. This result indicates that the native form of the protein exists in at least two multimeric states.

**Figure 9:**
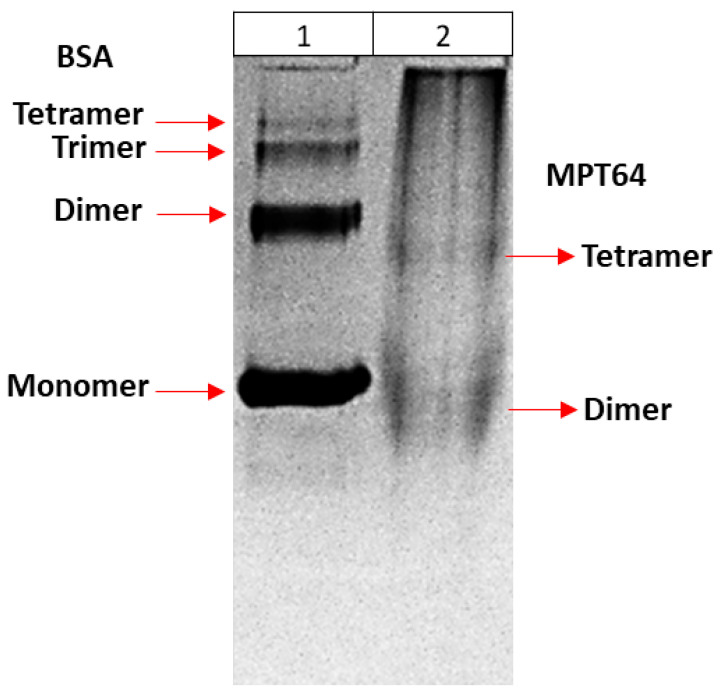
Native forms of recombinant MPT64 protein. (1) BSA; (2) Recombinant MPT64 protein. 12% Native PAGE.

In order to better understand the multimeric forms of the recombinant MPT64 protein, they were run on 12% native PAGE (Figure 9). Bovine Serum Albumin (BSA) was used as a control. According to the gel result, the forms of BSA were detected as 4 bands: monomeric, dimeric, trimeric, and tetrameric as expected [26]. Recombinant MPT64 was seen as two bands. One of the bands was located right below the monomeric form of BSA (66 KDa), and the other right below the dimeric form (132 KDa). Since the recombinant MPT64 protein is about 25 KDa in size, it suggests that it exists as a dimeric form (about 50 KDa) and a tetrameric form (about 100 KDa). These results were consistent with the previous literature data [14].

## 4. Discussion

The MPT64 protein, a biomarker of *Mycobacterium tuberculosis*, is important in diagnosis [7,27,28]. It plays a critical role in differentiating MTC from NTM. Many diagnostic products have been developed for the detection of MPT64 antigens released by *M. tuberculosis* during infection [29]. There are also studies suggesting that MPT64 protein released by the bacteria is important in escaping the body’s immune system [9]. Therefore, these proteins may also be important targets for therapy. To understand the properties of these proteins and their potential for in vivo or in vitro uses, it is necessary to produce them recombinantly with high yield and purity. There are publications on the recombinant production of MPT64 protein, but detailed data on the optimization of culture conditions and purification methods are very limited [12,13,14,15,16,17,18,19,30,31]. In this study, optimization studies for the production of MPT64 protein in high purity and quantities and characterization of the protein are described in detail.

Firstly, the MPT64 gene of the bacterium was not directly amplified by PCR when compared to previous studies. This was due to codon incompatibility. In Gram (-) *E. coli* bacteria, expression of the MPT64 gene derived from Mycobacteria results in low efficiency, as the codon usage similarity between the two organisms is only 77%. This significantly affects the yield of the expressed protein. To date, no studies in the literature have reported the use of a gene block obtained via codon optimization for this gene. In a study reported by Kusuma et al. (2019) [16], the use of a synthetic gene is mentioned; however, it was not specified whether the gene was codon-optimized. Moreover, the article focused only on optimization of protein expression and did not include details on purification, refolding, or the final protein yield.

The production of MPT proteins in another species of mycobacterium, *M. smegmatis*, which eliminates the need for codon optimization, was reported by Roche et al. (1996) [11]. Moreover, in this study, MPT64 protein was secreted into the culture medium. Despite these advantages, the method presented several challenges. For instance, M. smegmatis required more specific culture conditions compared to *E. coli*. A rich medium such as 7H9 broth supplemented with 5% (w/v) bovine serum albumin, 2% (w/v) glucose, and 0.03% (w/v) catalase was necessary, in contrast to the conventional media typically used for recombinant protein production in *E. coli*. Additionally, *M. smegmatis* required 3-day cultures for protein expression, which is considerably longer than the culture time for *E. coli*. Furthermore, LAM molecules, which are part of the cell wall structure of the pathogenic *M. tuberculosis*, are also present in *M. smegmatis*. Therefore, additional precautions must be taken during the purification process to ensure the removal of these LAM molecules. This is particularly important if the recombinant proteins are intended for in vivo use, as the presence of LAM antigens could trigger undesired immune responses.

For these reasons, the production of MPT64 protein in *E. coli* may be an important option. For this purpose, in our study, a codon optimization was first performed, and a gene block was designed subsequently. In addition, *E. coli* BL21(DE3)-R3-pRARE2 strain, which enables expression of rare codons, was used as a host for expression. Unlike previous studies that applied conventional cloning methods using restriction enzymes, in this study, the MPT64 gene was cloned by the LIC method [20]. This method required only one restriction enzyme digest (SspI) to linearize the plasmid vector, and no other restriction enzyme was required. The primers were designed to contain the LIC sites and could be easily added to the gBlock by PCR and cloned after treatment with the T4 DNA polymerase enzyme.

The other parameter investigated in this study was the contents of the culture media used for the production of the MPT64 protein. In our previous studies, it was shown that different media contents influenced the recombinant production of MPT64 protein [18]. Even though codon optimization was performed and a host having rare codon expression was used here, media contents were still investigated to determine their influence on protein production. For this purpose, standard bacterial culture media were primarily compared: LB, MHB, and BHI. Of these three media, LB is the most common medium used in biotechnology laboratories for recombinant protein production. MHB is a standard medium used in microbiology laboratories for minimum inhibitory concentration (MIC) assays of almost all microorganisms. BHI is also used for the culture of microorganisms that are difficult to grow due to its very rich content. The sources of amino acids are yeast extract and tryptone in LB medium, beef and casein in MHB medium, and beef heart, calf brain, and peptone in BHI medium. In this regard, it is impossible to know the exact content of BHI and MHB, but we can have information about the amino acid content of tryptone/peptone and yeast extract in LB (Table 1) [32,33,34].

Interestingly, MHB suppressed the multiplication of bacteria containing the MPT64 gene unlike BHI, although the content of MHB is similar to BHI. The slowing of bacterial growth was also observed before induction of expression by IPTG, suggesting that the effect of MHB is not solely dependent on the expression of the MPT64 gene.

The results obtained in this study showed that three different media had a similar effect on the expression of MPT64 protein in *E. coli*. MHB medium was eliminated due to its inhibitory effect on the growth of these bacteria. The studies were continued with BHI due to its richer content compared to LB medium. Furthermore, BHI medium was supplemented with MgCl_2_ and glycerol for higher protein production. Because magnesium was found to have an effect on the growth and division of various bacterial species in bacterial culture media and was shown to be essential for normal cell division, especially in rod-shaped bacteria [35]. Glycerol as a carbon source was shown to be advantageous over glucose for maximizing recombinant protein production as inclusion bodies in *E. coli* BL21(DE3) via lactose-induced systems [36]. In our study, a very significant increase in the production of recombinant MPT64 protein was observed upon the addition of MgCl_2_ and glycerol. These results are compatible with the positive effect of MgCl_2_ and glycerol reported in the literature [35,36]. In addition, protein production was maximized with the addition of peptone and tryptone (eBHI medium).

Using the optimized eBHI medium, an increased bacterial density (2.5 at OD_600_) before IPTG induction and expression time (approximately 24 h) were important to obtain a high yield of protein. Since recombinant MPT64 protein was produced as inclusion bodies, they had to be solubilized with denaturants such as urea and Gu-HCl after washing steps. Refolding of the protein was attempted by shock dilution but was not successful due to the rapid precipitation of proteins. However, the refolding of the protein on the column was successful thanks to binding the proteins to the nickel beads. Different imidazole concentrations were tested for elution of the protein, and the best results were obtained in the range of 100–200 mM imidazole.

The proteins obtained in this study were confirmed by using a lateral flow assay. In addition, multimeric forms of proteins in their native state were shown by using ultrafiltration and native PAGE. According to these results, the MPT64 protein had dimeric and tetrameric forms, which are compatible with the literature [14]. The alpha helix and beta sheet structures of the protein were also verified by the CD method as reported in the literature [37]. To the best of our knowledge, this study provides the most detailed information on the recombinant production and characterization of MPT64.

The yield of recombinant MPT64 protein in the literature is reported as 0.75 mg/L in *E. coli* and 3.8 g/L in *M. smegmatis* [12,13]. However, Geisbrecht et al. (2006) reported that the amount of protein produced in *E. coli* was 250 mg/L after purification using scalable anion-exchange chromatography and gel filtration chromatography [13]. In our study, the amount of recombinant MPT64 protein was 4.9 mg/L, with “L” representing one liter of culture medium. Since purification systems used in the purification steps of Geisbrecht et al. (2006) such as reversed-phase and size exclusion HPLC are sophisticated and relatively expensive equipment, the use of simple nickel columns in our study may be a more convenient and efficient way for research laboratories [13].

In conclusion, the MPT64 protein was produced in *E. coli* in high yield and purity as a result of the gBlock design and all these optimization studies. These results provide important data for future studies on the MPT64 protein.

## Figures and Tables

**Table 1:** Comparison of amino acid contents of culture media additions and the MPT64 protein.

	(%)			
Aminoacids	MPT64	Peptone	Tryptone	Yeast Extract
Leucine	9.6	10.99	7.7	3.8
Alanine	7.4	6.9	2.8	4.4
Proline	6.8	2.84	9.1	2
Lysine	6.4	7.19	7	4
Aspartic acid	6.2	9.93	6.7	4.9
Glycine	6.2	4.06	1.8	2.4
Isoleucine	5.7	0.15	4.7	2.7
Serine	5.7	3.88	5.1	2.3
Threonine	5.7	2.47	3.9	2.1
Tyrosine	5.7	1.92	1.3	1.4
Glutamic acid	5.5	7.25	18	8.1
Valine	5.3	6.79	6	2.9
Glutamine	4.7	-	-	-
Phenylalanine	3.8	5.93	4.1	2.3
Asparagine	3.4	-	-	-
Arginine	3.2	3.22	3.2	2.5
Methionine	3	0.71	2.5	0.9
Histidine	2.8	6.34	2.4	1
Cystine	1.7	<0.1	0.3	0.4
Tryptophan	1.3	1.25	1	0.6

## Data Availability

The datasets collected and analyzed during this study are not available in the public domain; however, they can be obtained from the corresponding author after reasonable request.

## Supplementary Materials

The following supporting information can be downloaded at: https://www.mdpi.com/article/10.3390/life15091472/s1, Table S1: Primers used for adding LIC sites on gBlock; Figure S1: MPT64 gene with codon optimization; Figure S2: PCR amplicons at different binding temperatures (1.5% agarose gel); Figure S3: PCR amplicons at different concentrations of DMSO (1.5% agarose gel); Figure S4: Treated plasmids with SspI enzyme (0.8% agarose gel); Figure S5: Colony screening by PCR (1.5% agarose gel); Figure S6: The amino acid sequence of the MPT64 protein; Figure S7: Production of MPT64 proteins in LB medium (12% SDS PAGE); Figure S8: Production of MPT64 proteins in MHB medium (12% SDS PAGE); Figure S9: Production of MPT64 proteins in BHI medium (12% SDS PAGE); Figure S10: Production of MPT64 proteins in sBHI medium (12% SDS PAGE); Figure S11: Production of MPT64 proteins in eBHI medium (12% SDS PAGE); Figure S12: Optimization of imidazole concentrations.
