# Supplementary material for: Expression of a Tuberculosis-Associated Immunogenic Protein in Escherichia coli
Source: Life (Basel). 2025 Sep 19;15(9):1472. doi: 10.3390/life15091472 (PMC12471812; doi:10.3390/life15091472)
Supplement: Supplementary file 1 [file life-15-01472-s001.zip › life-3813234-supplementary.pdf]

Supporting Document

Expression of a Tuberculosis-Associated Immunogenic Protein in *Escherichia coli*

Gizem Kılıç[1], Burcu Saygıner[1], Muhammed Yusuf Yılmaz[1], Bilge Suyolcu[1], Neda Tatlıoğlu[1], Ayça Tan[1], Tanıl Kocagöz[1,2], Nihan Ünübol[1,3], Erkan Mozioğlu[1*]

[1]Department of Medical Biotechnology, Institute of Health Sciences, Acibadem University, İstanbul, Türkiye

[2]Department of Medical Microbiology, Faculty of Medicine, Acibadem University, İstanbul, Türkiye

[3]Medical Laboratory Techniques, Vocational School of Health Services, Acibadem University, İstanbul, Türkiye

[*] Corresponding author. E-mail: erkanmozioglu@yahoo.com ; erkan.mozioglu@acibadem.edu.tr

Design of Gene block (gBlock) and PCR Optimisation

The immunogenic MPT64 protein sequence of *M. Tuberculosis*, which is 228 amino acids in length, was obtained from NCBI (NP_216496.1). The pI of the MPT64 protein is 4.84. In order to determine the most likely possible DNA sequences that allow this protein to be produced in *E. coli*, codon optimization was performed. Sequences with codon optimization were purchased as gBlock from IDT Inc. *pET His6 TEV LIC* was used as cloning and expression vector, which was developed by Scott Gradia and deposited in Addgene (# 29653). Cloning steps into this vector followed the instructions recommended by Scott Gradia.[20] Briefly, LIC primers were designed according to gBlock as given in Table S1, and LIC sites (shown in capital letters and bold) were added at the 5' end of both forward and reverse primers:

Table S1: Primers used for adding LIC sites on gBlock

Primers	Sequences (5' to 3')
Forward	**TACTTCCAATCCAATGCA**atgcgtatcaaaatcttcatgc
Reverse	**TTATCCACTTCCAATGTTATTA**tgccagcatgctatcaat

gBlock was bought as 500 nmol and resuspended according to the manufacturer's instructions. Briefly, it was dissolved in 50 µL of apyrogenic water, resulting in 10 ng/µL. After vortexing, the tube was kept at 50°C for 20 minutes. The DNA sample was then serially diluted and stored at -80°C until used.

For PCR, firstly, different binding temperatures (68, 70, and 72°C) were tested, and the reaction mixture was prepared as follows: A final concentration of 0.2 mM dNTP, 0.5 mM primers and 0.02 U Phusion High-Fidelity DNA Polymerase, 20 ng gBlock in 1X GC buffer (NEB). PCR conditions were as follows: 98°C for 3 min for initialization, 98°C for 10 s, 68/70/72°C for 30 s, 72°C for 30 s for 30 cycles, and then 72°C for 10 min.

Different concentrations of DMSO (3%, 4%, 5%, and 6% (v/v)) were added to the PCR mix to remove nonspecific amplification products. PCR conditions were as follows: 98°C for 3 min for initialization, 98°C for 10 s, 72°C for 90 s for 30 cycles, and then 72°C for 3 min.

Ligation Independent Cloning (LIC) and Transformation

Bacteria having plasmids were amplified in LB media containing 50 µg/mL kanamysin, and plasmids were purified by using Plasmid Purification Kit (GeneMark). Then, plasmids were linearized by SspI enzymes at 37ºC for 3 hours and 65ºC for 15 minutes.[20] Both plasmid vectors and gBlock-PCR products were sticky-ended by the T4 polymerase enzyme.[20] Since the T4 polymerase enzyme shows exonuclease activity in the presence of only a single nucleotide, PCR products were treated with dCTP, while plasmid vectors were treated with dGTP. The reaction was as follows: 22ºC for 30 minutes and 75ºC for 20 minutes. Then, both products were mixed in different ratios and kept at 70ºC for 1 minute, 22ºC for 5 minutes, and on ice for 30 minutes.[20]

Transformation was performed by applying heat shock to bacteria that were made competent by the $CaCl_2$ method. For this purpose, 50 µL of competent bacteria (DH5α) and cloned plasmids were kept together on ice for 10 minutes. Then, the tubes were incubated at 42°C for 90 s and kept on ice for 2 minutes. 450 µL of liquid LB medium was added to the tubes and incubated at 37°C for 1 hour. Then, bacteria were spread on LB agar medium containing 50 µg/mL kanamycin sulfate. Petri dishes were incubated at 37°C overnight, and colonies were confirmed by PCR. Plasmids were purified from colonies confirmed by PCR, and sequences were confirmed by Sanger Sequencing. The confirmed plasmids were transferred to *E. coli* BL21(DE3)-R3-pRARE2 strain having seven rare-codon tRNA genes for expression. Bacterial colonies having plasmids carrying the MPT64 gene were confirmed by colony PCR. For PCR, 5x PCR Master Mix (GeneMark), primers T7 promoter (TAATACGACTCACTATAGGG) and T7 terminator (GCTAGTTATTGCTCAGCGG) at a final concentration of 0.5 µM were used. PCR conditions were as follows: 95°C for 3 min for initialization, 95°C for 30 s, 53°C for 30 s, 72°C for 1 min for 30 cycles, and then 72°C for 5 min. Then, confirmed colonies were stocked and stored at -80ºC to be used for expression.

Optimization of requirements for recombinant protein expression

Optimization was first performed by comparing the effects of different medium ingredients on protein expression efficiency. For this purpose, 5 different culture conditions were investigated. The second parameter was IPTG; it was determined whether IPTG affects the expression under the specified culture conditions. Another parameter was the effect of time on expression. Since it is an important parameter to determine whether the protein is produced in soluble form or as an inclusion body for the next steps of extraction and purification, we finally determined where the protein was located in those culture conditions assisting the highest protein expression over time.

Different culture media used for protein expression experiments are as follows:

- **LB (Merck):** 1% (w/v) of tryptone, 0.5% (w/v) of yeast extract, 1% (w/v) of NaCl
- **MHB (Across Bio):** 2% (w/v) of beef extract, 0.15% (w/v) of starch, 1.75% (w/v) of casein hydrolysate
- **BHI (Across Bio):** 0.78% (w/v) of brain extract, 0.2% (w/v) of dextrose, 0.25% (w/v) of disodium phosphate, 0.97% of heart extract, 1% of proteose peptone, 0.5% sodium chloride
- **Supplemented BHI (sBHI):** (BHI including $MgCl_2$ of 0.03% (w/v) and 0.4 (v/v) of glycerol)
- **Enriched BHI (eBHI):** (sBHI including 0.15% (w/v) of peptone and 0.15% (w/v) of tryptone).

All media were supplemented with 50 µg/mL kanamycin sulfate and 25 µg/mL chloramphenicol for selectivity. Bacteria were grown as starter culture at 37°C at 180 rpm by shaking overnight. The next day, it was transferred to a large volume (200 mL) of fresh culture medium and allowed to grow under the same conditions. Once the optical density (600 nm) was 0.5, IPTG was added to the culture at a final concentration of 0.1mM, and 100 µL of the growing bacterial culture was taken every hour. The samples without IPTG were also taken as controls at the 0th hour, 5th hour, and 24th hour. The bacteria were precipitated by centrifugation at 16,000xg for 5 min, and the supernatant was removed. 500 µL of a loading buffer (2% (w/v) of SDS, 2 mM dithiothreitol, 4% (v/v) of glycerol, 0.01% (w/v) of bromophenol blue, 50 mM Tris-HCl buffer pH 6.8) was added to them. 10 µL of sample was run in 12% SDS gel.

Gels were stained with the CBB-HCl method.[21] Briefly, first CBB-HCl was prepared as follows: 60 mg of Commasie Brillant Blue G 250 was dissolved in 1 L of double-distilled deionized water and stirred with a magnet for 2 hours. 3 mL of concentrated HCl was added dropwise and stirred for 2 min. The polyacrylamide gel was first rinsed with water to remove SDS and then immersed in distilled water. The gel was heated in a microwave oven for 30 s and kept at room temperature for 5 min by gentle shaking. Then, the water was removed, and this washing step was repeated. After removal of the water, CBB-HCl was added to cover the gel and heated in a microwave oven for 15 sec. Then, it was shaken gently and waited until stained.

In order to determine the effects of bacterial density on total protein yield, eBHI medium was used, which gave the best results during optimization. For this purpose, eBHI medium was prepared as described above. Bacteria were grown as starter culture at 37°C at 220 rpm with shaking overnight. The next day, it was transferred to a large volume of fresh culture medium and allowed to grow under the same conditions. Once the optical density at 600 nm was 0.6 and 2.5, IPTG was added to cultures at a final concentration of 0.1 mM, and bacteria were grown for 22 hours under the same conditions. The bacteria were precipitated by centrifugation at 10,000xg for 10 min and then suspended in 5 mL of 10 mM Tris-HCl buffer (pH 8.0) including 500 µg lysozyme and 100 µg DNAse I. The sample was kept at room temperature for 30 min by shaking, and then ultrasonic sound was applied for 5 minutes. After centrifugation at 10,000xg for 1 hour, the supernatant was removed and the pellet was dissolved in 5 mL of 7M urea for 40 minutes at room temperature. Both the supernatant and the precipitate were analyzed using 12% SDS-PAGE.

Supporting Figures:

```
atgcgtatcaaaatcttcatgctggtgaccgcggtggtgctgctgtgctgcagcggcgtg
gcgaccgcggcgccgaaaacctattgcgaagaactgaaaggcaccgataccggccaggcg
tgccagattcagatgagcgatccggcgtataacattaacattagcctgccgagctattat
ccggatcagaaaagcctggaaaactatattgcgcagacccgcgataaatttctgagcgcg
gcgaccagcagcaccccgcgcgaagcgccgtatgaactgaacattaccagcgcgacctat
cagagcgcgattccgccgcgcggcacccaggcggtggtgctgaaagtgtatcagaacgcg
ggcggcacccatccgaccaccacctataaagcgtttgattgggatcaggcgtatcgcaaa
ccgattacctatgataccctgtggcaggcggataccgatccgctgccggtggtgtttccg
attgtgcagggcgaactgagcaaacagaccggccagcaggtgagcattgcgccgaacgcg
ggcctggatccggtgaactatcagaactttgcggtgaccaacgatggcgtgattttttt
tttaacccgggcgaactgctgccggaagcggcgggcccgacccaggtgctggtgccgcgc
agcgcgattgatagcatgctggca
```

Figure S1. MPT64 gene with codon optimization.

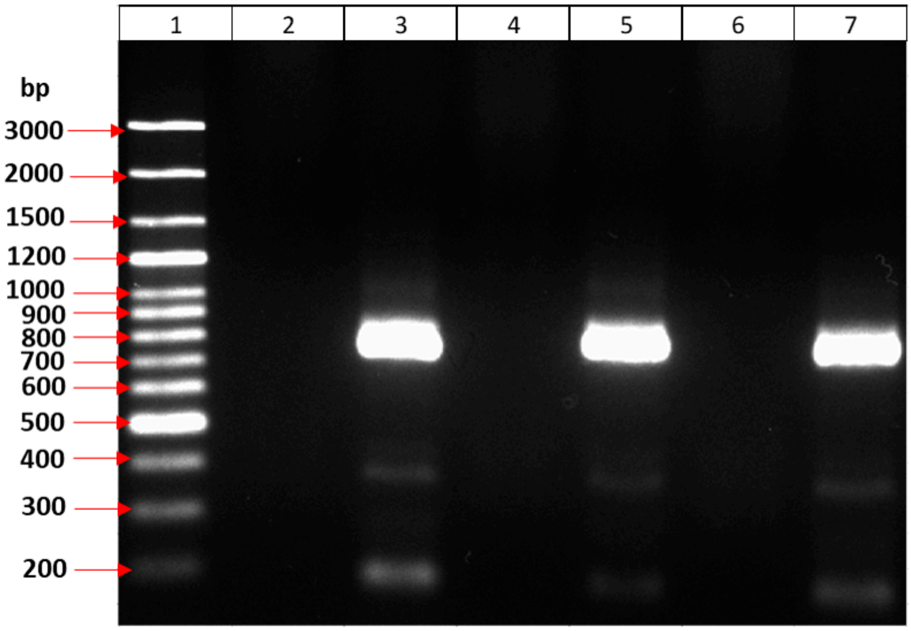

Figure S2. PCR amplicons at different binding temperatures (1.5% agarose gel). 1) Marker (100 bp, GeneMark); 2) no-template control (NTC) for 72ºC of Tm; 3) amplicon of gBlock for 72ºC of Tm; 4) no-template control (NTC) for 70ºC of Tm; 5) amplicon of gBlock for 70ºC of Tm; 6) no-template control (NTC) for 68ºC of Tm; 7) amplicon of gBlock for 68ºC of Tm.

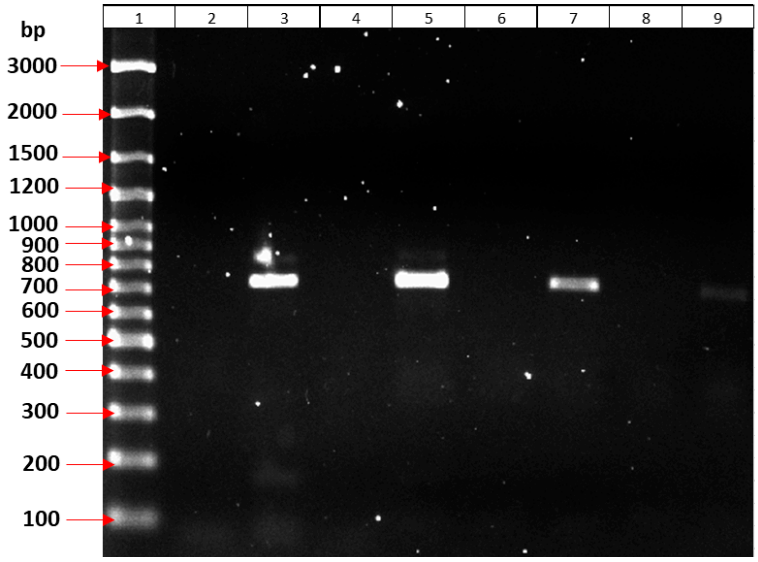

Figure S3. PCR amplicons at different concentrations of DMSO (1.5% agarose gel). 1) Marker (100 bp, GeneMark); 2) no-template control (NTC) in 3% of DMSO; 3) amplicon of gBlock in 3% of DMSO; 4) no-template control (NTC) in 4% of DMSO; 5) amplicon of gBlock in 3% of DMSO; 6) no-template control (NTC) in 5% of DMSO; 7) amplicon of gBlock in 5% of DMSO; 8) no-template control (NTC) in 6% of DMSO; 9) amplicon of gBlock in 6% of DMSO.

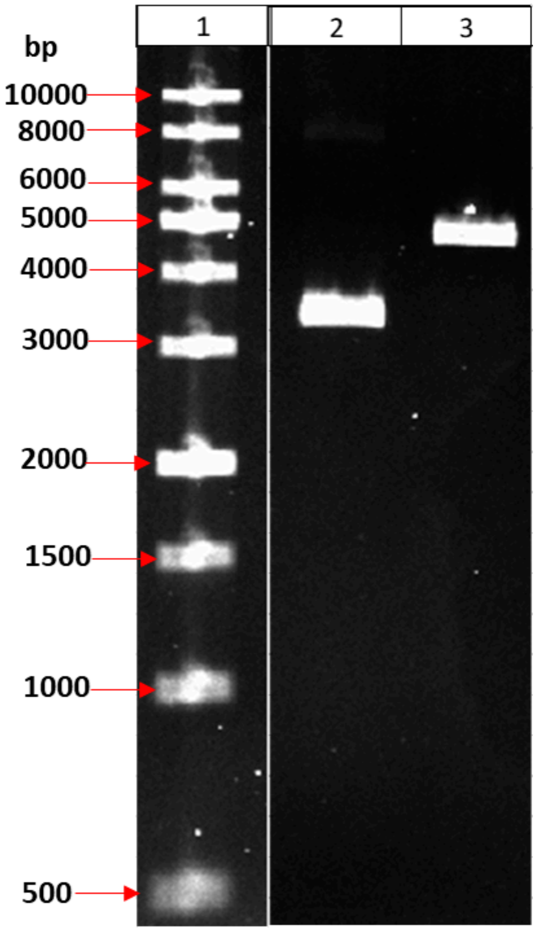

Figure S4. Treated plasmids with SspI enzyme (0.8% agarose gel). 1) Marker (1000 bp, GeneMark); 2) plasmid without enzyme treatment; 3) plasmid with enzyme treatment.

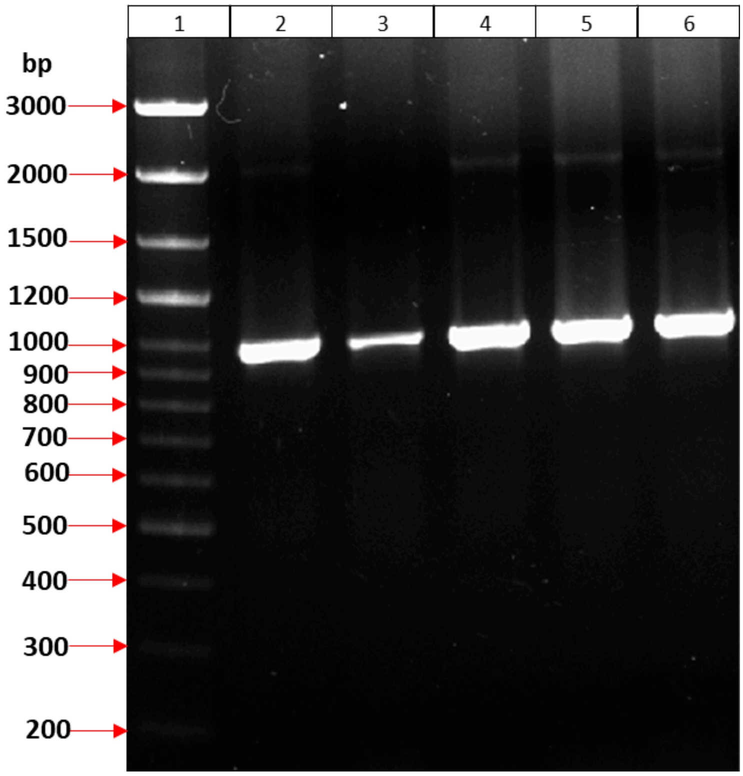

Figure S5. Colony screening by PCR (1.5% agarose gel). 1) Marker (100 bp, GeneMark); 2-6) Amplicons obtained by PCR from different colonies.

MRIKIFMLVTAVVLLCCSGVATAAPKTYCEELKGTDTGQACQIQMSDPAYNINISLPSYYPDQKSLEN
YIAQTRDKFLSAATSSTPREAPYELNITSATYQSAIPPRGTQAVVLKVYQNAGGTHPTTTYKAFDWDQ
AYRKPITYDTLWQADTDPLPVVFPIVQGELSKQTGQQVSIAPNAGLDPVNYQNFAVTNDGVIFFFNPG
ELLPEAAGPTQVLVPRSAIDSMLA

Figure S6. The amino acid sequence of the MPT64 protein.

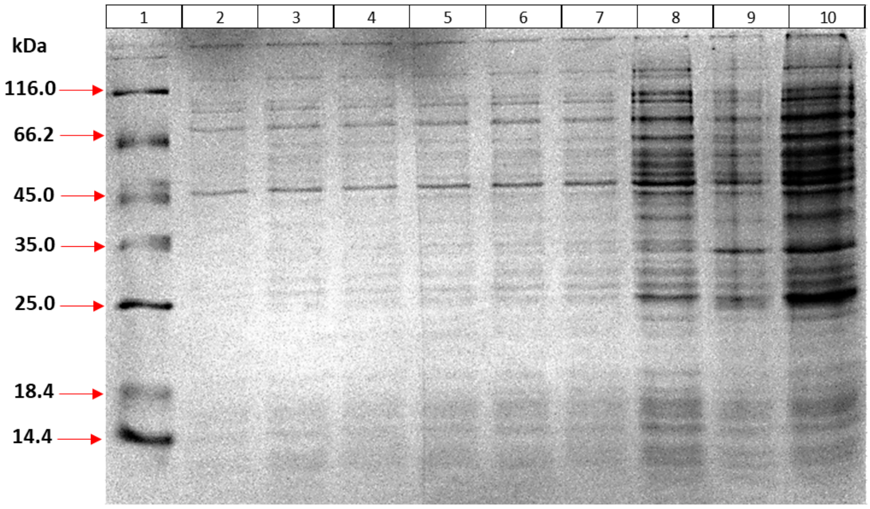

Figure S7. Production of MPT64 proteins in LB medium (12% SDS PAGE). 1) Marker; 2) Sample before IPTG addition; Sample at 3) 1st hour, 4) 2nd hour, 5) 3rd hour, 6) 4th hour, 7) 5th hour after IPTG addition; 8) Sample 5th hour without IPTG; Sample at 24th hour 9) after IPTG, and 10) before IPTG.

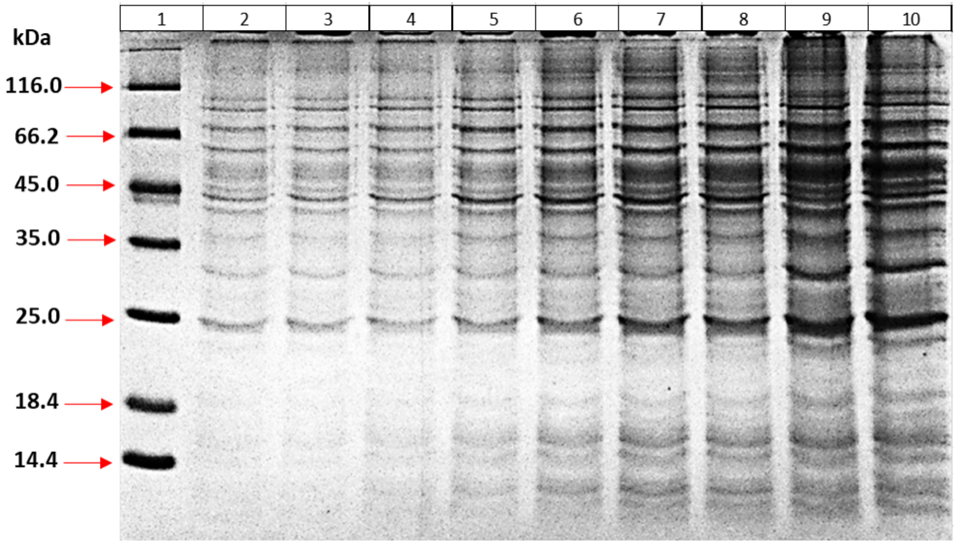

Figure S8. Production of MPT64 proteins in MHB medium (12% SDS PAGE). 1) Marker; 2) Sample before IPTG addition; Sample at 3) 1st hour, 4) 2nd hour, 5) 3rd hour, 6) 4th hour, 7) 5th hour after IPTG addition; 8) Sample 5th hour without IPTG; Sample at 24th hour 9) after IPTG, and 10) before IPTG.

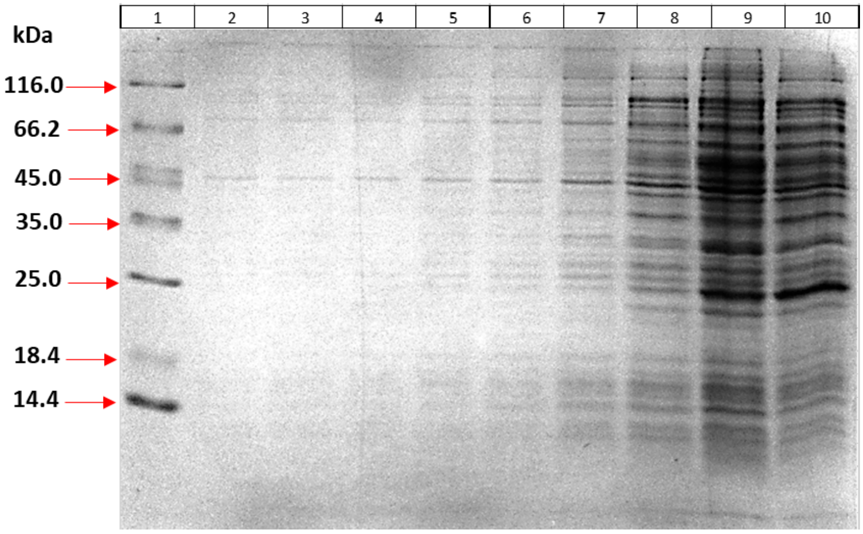

Figure S9. Production of MPT64 proteins in BHI medium (12% SDS PAGE). 1) Marker; 2) Sample before IPTG addition; Sample at 3) 1st hour, 4) 2nd hour, 5) 3rd hour, 6) 4th hour, 7) 5th hour after IPTG addition; 8) Sample 5th hour without IPTG; Sample at 24th hour 9) after IPTG, and 10) before IPTG.

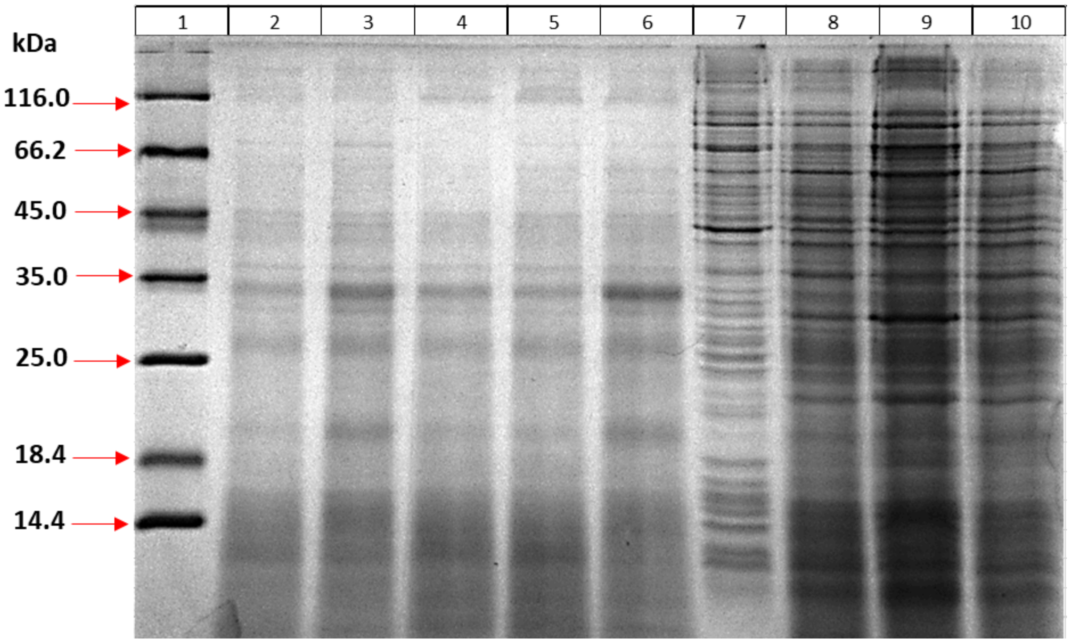

Figure S10. Production of MPT64 proteins in sBHI medium (12% SDS PAGE). 1) Marker; 2) Sample before IPTG addition; Sample at 3) 1st hour, 4) 2nd hour, 5) 3rd hour, 6) 4th hour, 7) 5th hour after IPTG addition; 8) Sample 5th hour without IPTG; Sample at 24th hour 9) after IPTG, and 10) before IPTG.

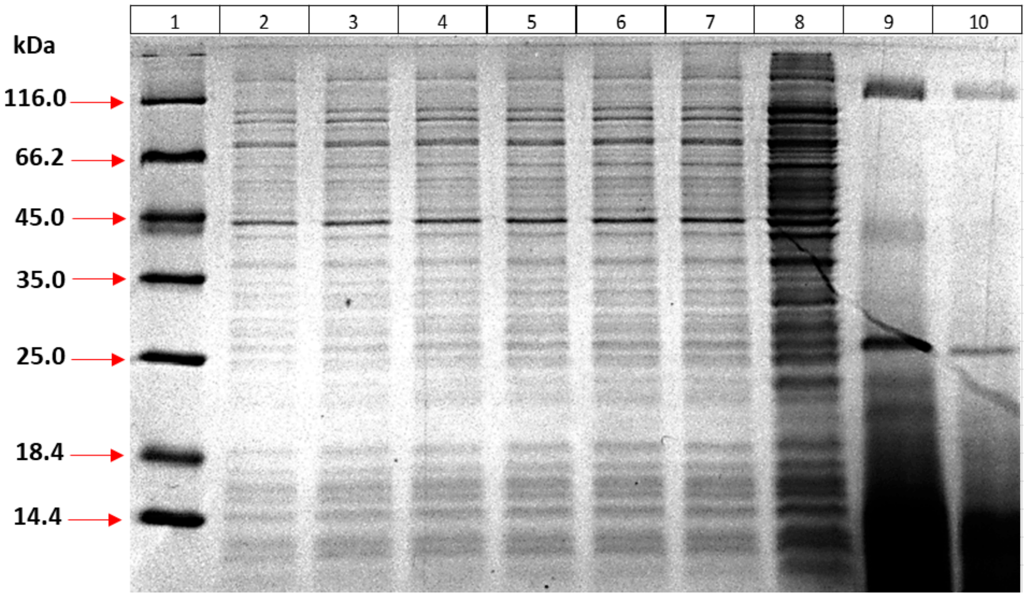

Figure S11. Production of MPT64 proteins in eBHI medium (12% SDS PAGE). 1) Marker; 2) Sample before IPTG addition; Sample at 3) 1st hour, 4) 2nd hour, 5) 3rd hour, 6) 4th hour, 7) 5th hour after IPTG addition; 8) Sample 5th hour without IPTG; Sample at 24th hour 9) after IPTG, and 10) before IPTG.

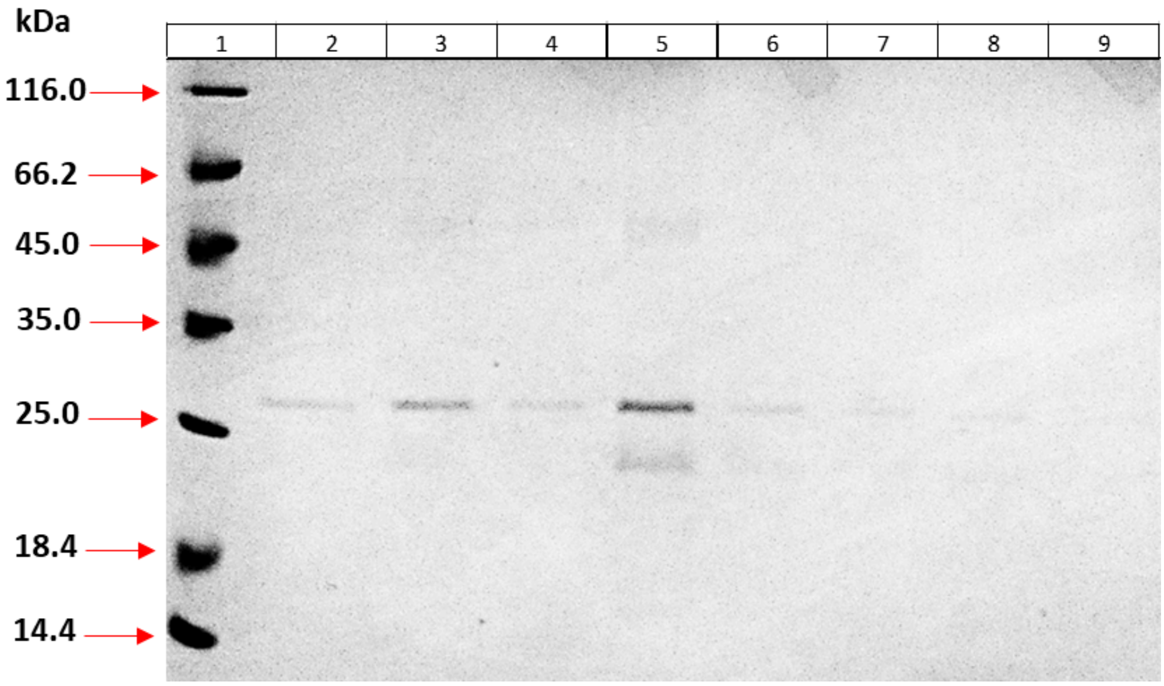

Figure S12. Optimization of imidazole concentrations. 1) Marker (Fermentas); Eluted proteins in buffer including 2–4) 100, 5) 200, 6) 250, 7) 350, 8) 500, and 9) 500 mM imidazole.
